# Exploring the Significance of the Obturator Externus Groove as a Marker for Bipedalism: A Cadaveric Study

**DOI:** 10.7759/cureus.109437

**Published:** 2026-05-22

**Authors:** Kamal A Abouzaid, Axel B Lichtenberg, Michael K Coffin, Ahmad Imam, Ahmad Y Karim, Jacob E Harwell, Amer A Khan, Logan S Rogers, Chiung K Wu, Emily R Stack, Mohammad K Algoul, Seyedeh Yassmin H Emami, Thienngan T Do, Danya Abouzaid, Robert Trigg

**Affiliations:** 1 Department of Anatomy, William Carey University College of Osteopathic Medicine, Hattiesburg, USA

**Keywords:** bipedalism, cadaveric study, comparative anatomy, evolutionary marker, femoral neck morphology features, human anatomy, obturator externus groove, phylogenetic femoral neck changes

## Abstract

Background

Bipedalism, defined as locomotion using only two feet, is a distinctive trait that sets humans apart from most other creatures. This study explores the significance of the obturator externus groove (OEG) as a marker for bipedalism. This topic is important due to its implications in evolutionary history. Previous studies have identified various anatomical features indicative of bipedalism, including an S-shaped spine, a centrally positioned foramen magnum, and unique pelvic and femoral structures. Among these, the morphology of the femur, particularly the presence of the OEG, has been proposed as a potential marker of habitual bipedalism.

Material and methods

This study examined the presence and dimensions of the OEG in both dry bone and cadaveric specimens. The dry bone study included 18 femur specimens from 14 individuals, analyzed by trained anatomists for the presence, visibility, and dimensions of the OEG. A cadaveric study was conducted on 28 formalin-fixed embalmed cadavers, utilizing precise dissection techniques to examine the relationship between the obturator externus tendon (OET) and the neck of the femur, and to identify and describe the OEG.

Results

In the dry bone study, 36% of individuals exhibited the OEG, while the cadaveric study found grooves in 21% of individuals. The OEG in this study was seen in 11 out of all 42 study subjects (dry bones and cadaveric), resulting in an incidence of about 26%. The groove’s depth varied, with shallow grooves measuring less than 1 mm and deeper grooves measuring more than 1 mm. Additionally, the study identified the obturator externus notch and a soft tissue bed cushioning the OET, providing new anatomical insights.

Conclusions

The findings revealed that the OEG is uncommon and typically observed unilaterally. The presence of the groove may result from biomechanical factors, such as activities requiring strong hip extension, rather than bipedal locomotion alone. The cadaveric dissection approach allowed for direct observation and measurement of the groove to minimize user interpretation bias, providing more accurate findings. The OEG was an infrequent finding, indicating that it should not be used as a criterion for determining bipedalism in fossils.

## Introduction

Bipedalism has been defined as locomotion using only two feet [1,2] and is a distinctive trait that sets humans apart from most other creatures [3]. The intriguing implications of this characteristic in evolutionary history have captivated the scientific community, sparking considerable interest among researchers aiming to understand the origins of mankind and unravel the genesis of bipedalism [4]. Among the numerous proposed indicators, several noteworthy features contribute to the identification of bipedal creatures in fossilized skeletons. These include an S-shaped spine that aids in balancing the upper body over the pelvis, a centrally positioned foramen magnum that aligns with the center of gravity [5], and a unique pelvic structure, particularly the ilium and the orientation of the hip joint, that supports the weight of the upper body during walking [6]. Furthermore, the morphology of the femur, including the position of the lesser trochanter, the height of the greater trochanter, and the obturator externus groove (OEG), are several characteristics that potentially suggest habitual bipedalism [7]. These characteristics collectively shed light on the multifaceted nature of bipedalism and provide valuable insights into the distinctive adaptations required for effective bipedal locomotion in various creatures.

The obturator externus muscle originates from the external surface of the obturator foramen and membrane and inserts into the trochanteric fossa [8]. The obturator externus is believed to be activated in motions related to external rotation of the hip [9,10]. Previous studies found that the obturator externus muscle also functions as a hip adductor when in certain positions [8,10]. Depending on the position of the obturator externus muscle against the femoral neck, it is believed to form the obturator externus groove (OEG). The OEG on the femoral neck has been proposed as a marker of bipedalism [7,9,11,12]. This hypothesis originated from a study on fossils in Tanzania, where the OEG was noted on a femur [11]. According to this study, the OEG formation is attributed to the consistent pressure of the obturator externus muscle tendon (OET) against the femoral neck during hip hyperextension.

The significance of the OEG in bipedalism is underscored by its presence in bipedal fossils such as *Homo erectus* and Rhodesian, contrasted with its absence in quadrupeds like African apes [11,13]. These studies suggest that the OEG is only present in truly bipedal creatures, like modern humans. One study concluded that thigh extension is greatest during climbing, allowing the obturator externus muscle to meet with the femoral neck, which was the most likely reason to develop an obturator externus groove over time [9]. However, there is conflicting evidence regarding these claims. According to a few studies, the obturator groove is present in some species of non-human primates that are not bipedal [9,14,15]. Furthermore, the scientific community has not reached a consensus on the definition or naming convention for the OEG, with various terms being used to describe it [11,14,16-20]. Variation in the naming terminology has led to confusion about the significance and prevalence of the groove in humans and early fossils.

This study is unique as it is the first to be conducted by trained anatomists on cadaveric specimens. Using a human model, this study aims to determine the incidence of the obturator externus groove on the femoral neck and evaluate its validity as a marker for bipedalism.

## Materials and methods

Dry bone study

**Figure 1:**
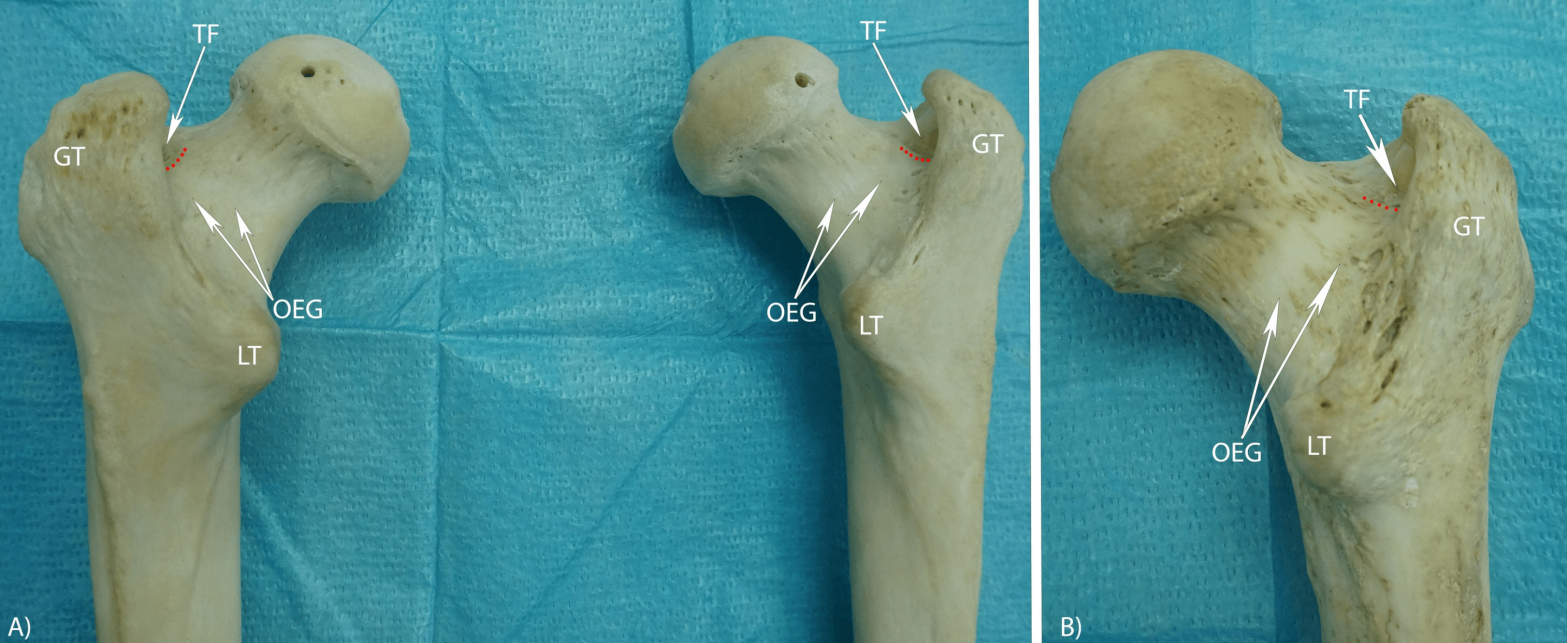
Posterior views of the proximal ends of femurs: The obturator externus groove was visualized on dry bone femur specimens. Image A shows the grooves that were on the femurs bilaterally, and image B shows a groove present in one of the unilateral femur specimens. OEG: obturator externus groove, GT: greater trochanter, LT: lesser trochanter, TF: trochanteric fossa Red dotted line: obturator externus notch

The dry bone study included human femurs that were available in the anatomy laboratory collection at William Carey University College of Osteopathic Medicine. Dry bone specimens were included if they were intact and free from significant deformity or damage that could interfere with identification or measurement of the obturator externus groove. We identified 10 unilateral femurs (7 right and 3 left) from 10 different individuals, and bilateral femurs from 4 individuals (articulated skeletons). These femur specimens were analyzed for the presence and dimensions of the obturator externus groove (Figure 1), and whether the groove was palpable or non-palpable. Then, the length, depth, and width of the obturator externus grooves were measured and analyzed.

Cadaveric study

**Figure 2:**
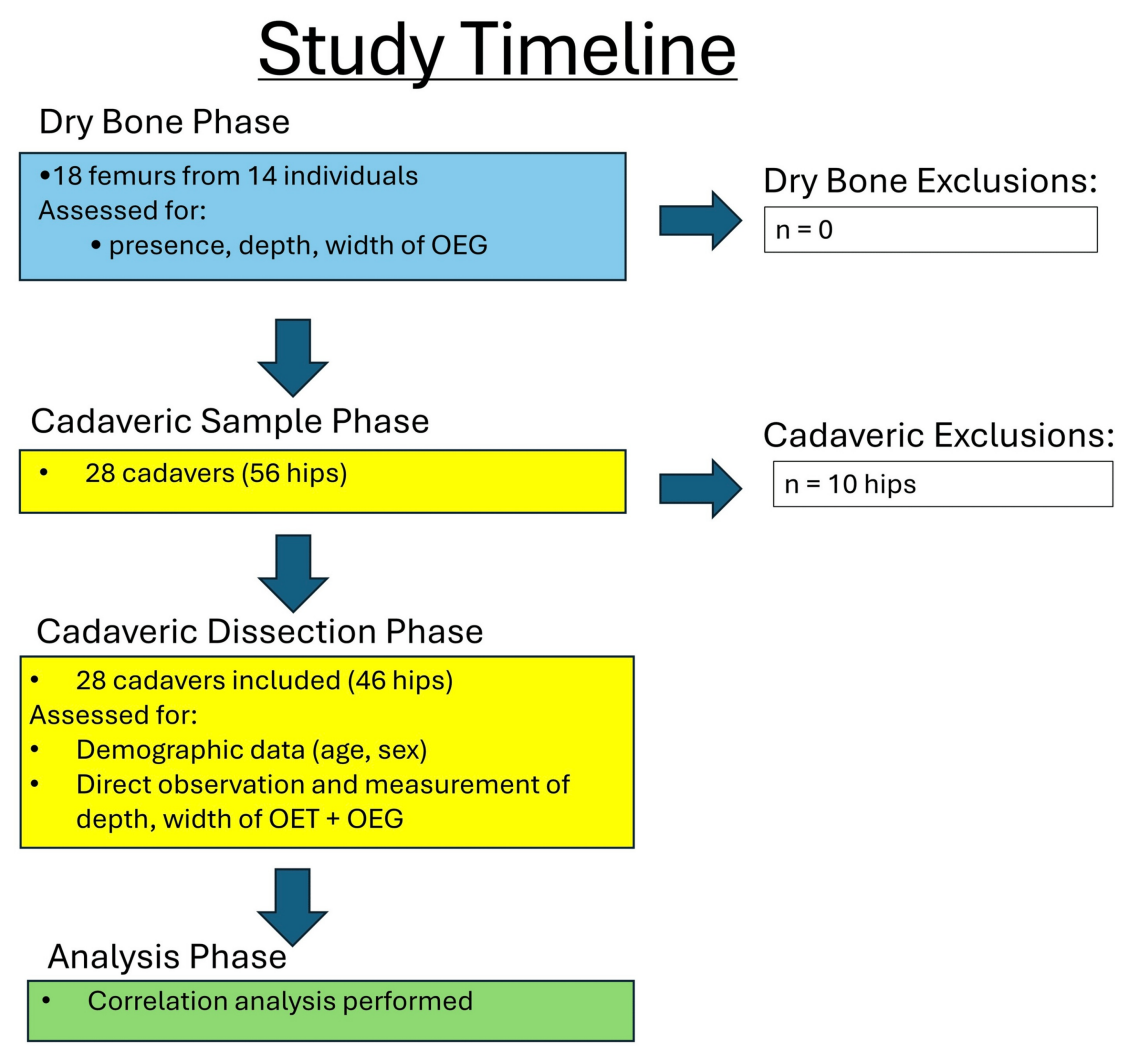
Study timeline illustrating the sequence of the dry bone analysis, cadaveric dissection phase, and statistical analysis. *Exclusions included prior dissection, orthopedic hardware, and hip replacement. OET: obturator externus tendon, OEG: obturator externus groove The figure was created by the authors using Microsoft PowerPoint version 2604 (Microsoft Corp., Redmond, WA).

Bilateral hip regions were dissected in 28 adult formalin-fixed embalmed cadavers at the Ross Anatomy Lab at William Carey University College of Osteopathic Medicine. The cadavers consisted of 15 male and 13 female donors. These cadaveric donors were obtained from the University of South Alabama Anatomical Gift Program, generously providing their bodies for anatomical education and research purposes. Cadaveric specimens were included if the hip region was intact and had not been previously dissected. Specimens with prior surgical intervention (e.g., hip replacements and intramedullary nails) or prior dissection were excluded to ensure anatomical integrity and accuracy of measurements. Of the total 56 hips that were dissected, 10 were excluded from the study due to previous surgeries (hip replacements and intramedullary nails) or due to prior hip joint dissections by medical students (Figure 2).

Dissection procedure

Starting with the cadaver in the supine position, the sartorius muscle was detached from the anterior superior iliac spine (ASIS) and reflected distally. Next, the rectus femoris muscle was detached from the anterior inferior iliac spine (AIIS) and retracted inferiorly. The femoral neurovascular bundle was then transected at the level of the inguinal ligament, followed by its inferior reflection. The iliopsoas muscle was detached from the lesser trochanter and reflected superiorly until the anterior capsule of the hip joint was visualized. The pectineus muscle was transected both superiorly and inferiorly and removed from the dissection field. At this point, attention was turned toward the obturator externus muscle, which was examined without disturbing its anatomy. The iliofemoral and pubofemoral ligaments were examined, and any muscle fibers attached to them were cleaned.

**Figure 3:**
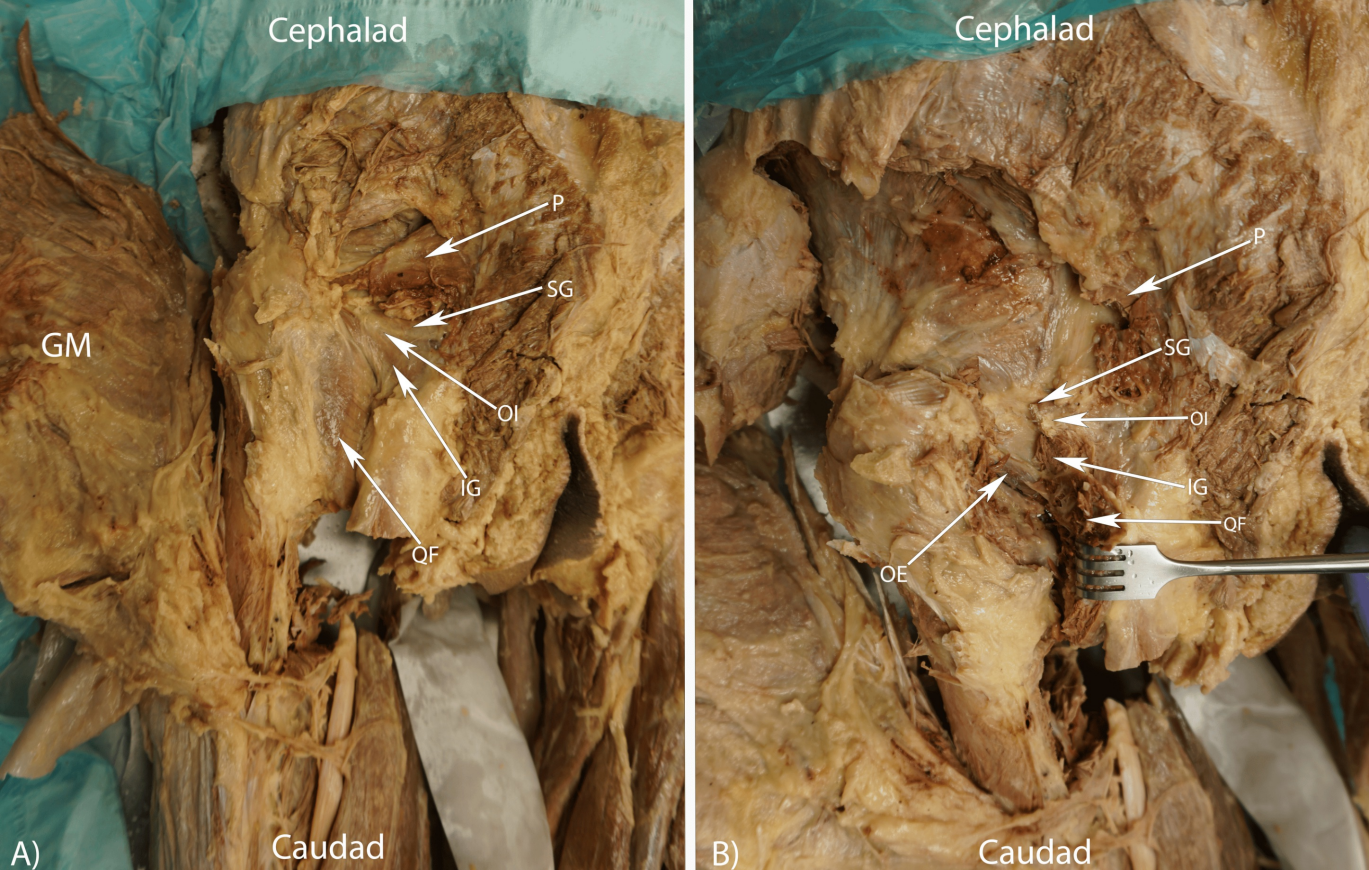
Posterior view of the left gluteal region: Image A shows the right short lateral rotator muscles prior to their resection, and image B shows the right short lateral rotator muscles after their resection to allow visualization of the OET tendon on the posterior aspect of the femur. GM: gluteus maximus, P: piriformis, SG: superior gemellus, OI: obturator internus, IG: inferior gemellus, QF: quadratus femoris, OE: obturator externus, OET: obturator externus tendon

The cadaver was then placed in the prone position, and the glutei maximus, medius, and minimus were transected from their femoral attachments and reflected superiorly and laterally. The piriformis, superior gemellus, obturator internus, inferior gemellus, and quadratus femoris muscles were detached from their attachment points on the femur and completely removed from the dissection field. The vessels and nerves passing superior and inferior to the piriformis muscle were removed to clear the field. Muscle fibers were cleared from the joint capsule and the ischiofemoral ligament. The tendon of the obturator externus was identified and examined in relation to the neck of the femur from a posterior view (Figure 3).

**Figure 4:**
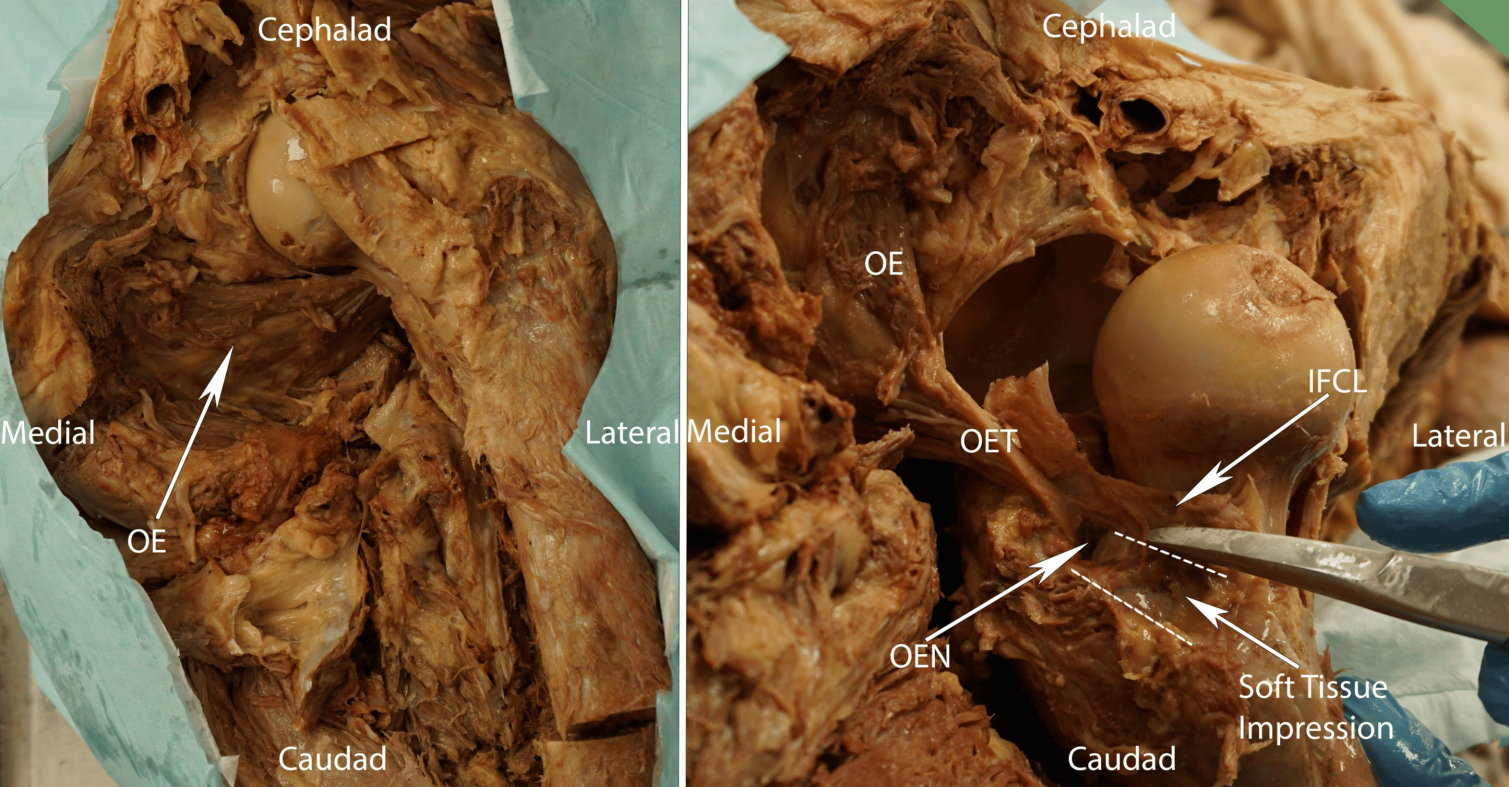
Anterior view of the left hip region: Image A (left) shows the obturator externus muscle. Image B (right) shows the limb abducted and laterally rotated to dislocate the femoral head to examine the obturator externus tendon, soft tissue impression, and ischiofemoral capsular ligament. OE: obturator externus, OET: obturator externus tendon, OEN: obturator externus notch, IFCL: ischiofemoral capsular ligament

The cadaver was then returned to the supine position, and the iliofemoral and pubofemoral ligaments and the joint capsule were opened through a transverse incision at the base of the neck, parallel to the intertrochanteric line and a second incision at a right angle with the transverse one. The lower limb was abducted and laterally rotated to expose and transect the ligament of the head of the femur. The femur was then rotated laterally to dislocate the head anteriorly. Muscles attached to the proximal shaft were stripped inferiorly to the midshaft of the femur, and the femur was then osteotomized at the junction of the upper and middle thirds of the shaft using a bone saw. Lateral rotation was performed again to examine the OET and its relationship to the neck of the femur, as well as the relationship between the ischiofemoral ligament and the neck of the femur (Figure 4).

**Figure 5:**
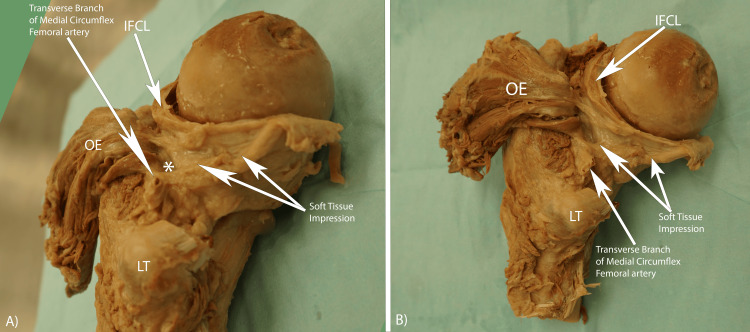
Posterior view of the proximal end of the left femur: The obturator externus muscle is retracted laterally to expose the soft tissue impression and fat overlying the obturator externus notch. *Location of the obturator externus notch OE: obturator externus, IFCL: ischiofemoral capsular ligament, LT: lesser trochanter

The obturator externus (OE) muscle was then sharply detached from its origin at the external surfaces of the obturator foramen and the obturator membrane. The iliofemoral, pubofemoral and ischiofemoral ligaments were then incised at their attachments to the hip bone. The proximal femur and the attached OE and the capsular ligaments were then removed and isolated from the cadaver, without disturbing the remaining soft tissue deep to the OET (Figure 5).

**Figure 6:**
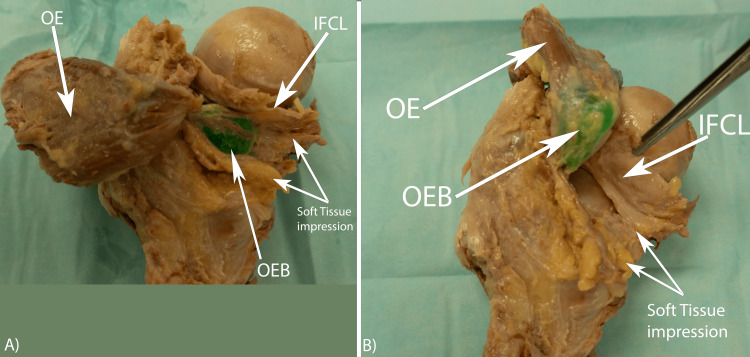
Posterior view of the left femur: The obturator externus is laterally retracted to expose the bursa. Image A shows the bursa in situ. Image B shows the bursa retracted to show the soft tissue impression. OE: obturator externus, IFCL: ischiofemoral capsular ligament, OEB: obturator externus bursa

To better understand the obturator externus bursa (OEB) and its relationship to the OET and soft tissue impression. The bursae of four specimens were infused with stained gelatinous material to delineate its borders proximally and distally, visualize its relation to the tendon, and recognize any possible communication with the hip joint cavity (Figure 6).

**Figure 7:**
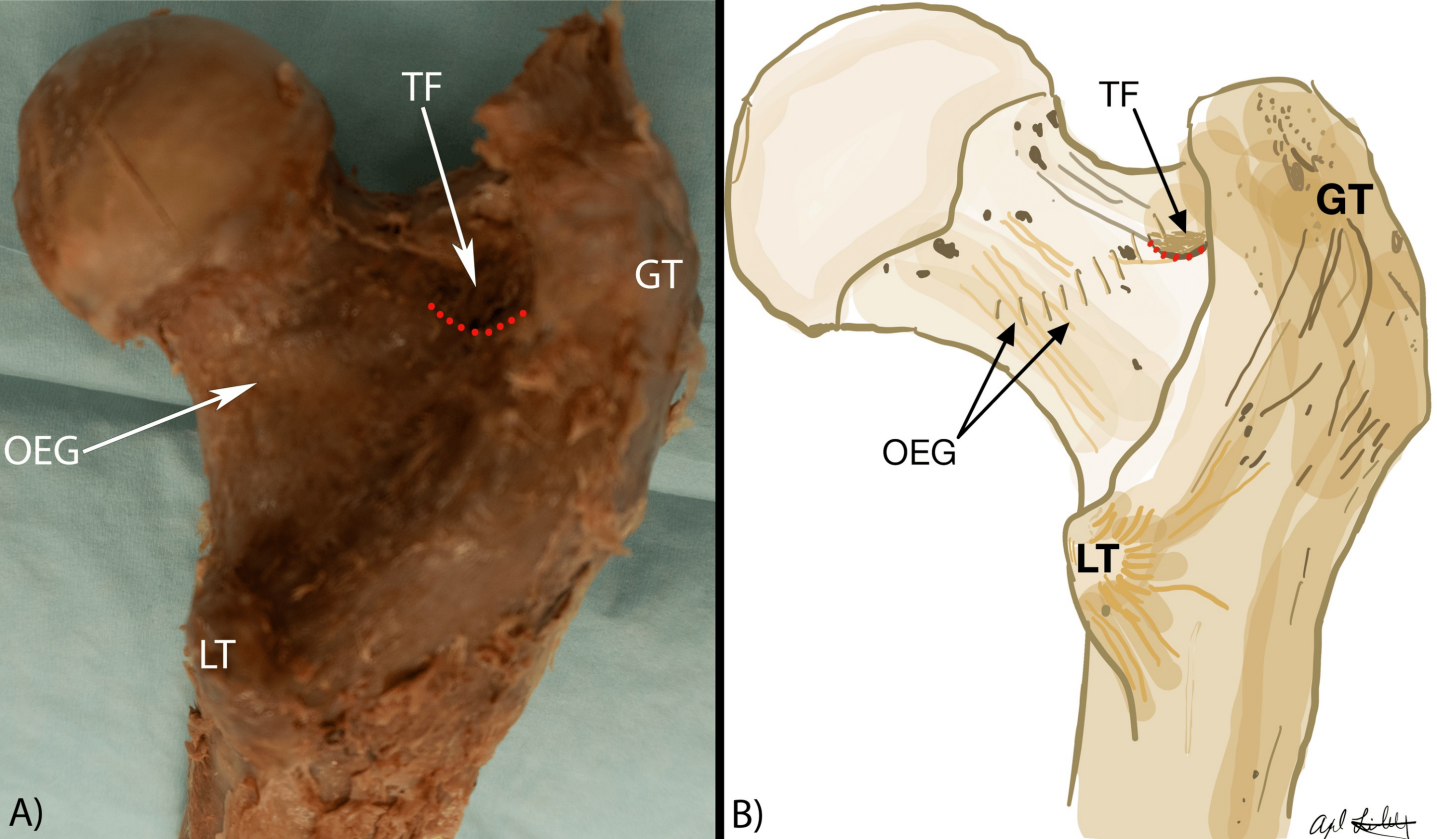
Posterior view of the right femur: The soft tissue and periosteum were cleaned (A) to allow visualization and measurements of the obturator externus groove. Image B shows a schematic representation for comparison. OEG: obturator externus groove, GT: greater trochanter, LT: lesser trochanter, TF: trochanteric fossa Red dotted line: obturator externus notch Image B is a comparison schematic drawn by one of the authors, Axel Lichtenberg, using Notability and was created without the use of AI.

At this point, the transverse branch of the medial circumflex femoral artery, the obturator externus bursa, and the ischiofemoral capsular ligament were removed from the posterior aspect of the femoral neck, and the tendon was cut as close as possible from its site of insertion on the trochanteric fossa. The posterior surface of the femur was then examined to identify the obturator externus notch, which is located at the junction between the greater trochanter and the neck of the femur (Figure 7). The capsule and periosteum over the posterior aspect of the neck of the femur were then cleaned to allow accurate measurements of the length, depth, and width of the groove (Figure 7). Upon identification and palpation of the OEG, the specimen was isolated.

Measurements of the obturator externus groove

The measuring tool used was the NEIKO 01408A 8” Electronic Digital Caliper (Neiko Tools, Gardena, CA) with precision and accuracy (measurement range: 0-8” and 0-200 mm, resolution: 0.0005”/1/128”/0.01 mm, accuracy: 0.001”/0.02 mm).

The measuring tools employed in this study were selected to ensure precision, accuracy, and reliability in obtaining measurements of the OEG. As was feasible, the same researchers were used to take each measurement to ensure they were taken as consistently as possible. The caliper was used for the length, depth, and width measurements of all femur specimens in this study. To ensure precise measurements, the instruments were zeroed and calibrated properly before recording each of the measurements. Using the caliper external measuring jaws, the length and width of OEG were measured.

Four anatomical experts examined all femurs and were required to reach a consensus on the presence of a visible groove and its palpability, attempting to replicate and improve upon the methodology described in the study by Lovejoy et al. [13]. An OEG was only considered “palpable” if all four experts agreed they visualized and felt a palpable OEG extending medially from the obturator externus notch. The length of the groove was then measured from the obturator externus notch to the point where the groove terminated on the posterior surface of the neck of the femur. Next, the edges of the groove were identified, and the width was measured at the midpoint using the caliper jaws. To measure the depth of the groove, the base of the main scale was placed to rest on the edges of the groove at the midpoint lengthwise. The depth gauge was advanced until it touched the bone cortex at the floor of the groove. The grooves were then categorized as shallow or deep based on the depth, where a groove depth of less than 1 mm was shallow and greater than 1 mm was deep.

The length of the OET was measured to see if tendon length correlates with groove length. To measure the length of the main tendon, one of the caliper jaws was placed distally at the cut end where the insertion was removed from the trochanteric fossa, and the other jaw was placed proximally at the end of the muscle-tendon junction, where no muscle fibers were integrated with the tendon.

Statistical analysis

All measurements were recorded on a data sheet at the time of measurement. Correlational analysis was done comparing obturator externus groove length to obturator externus tendon length, and a correlation coefficient was calculated. Groove length, depth, and width were expressed as a range and mean ± SD. Statistical significance was set at p < 0.05. The calculations and analysis were done using IBM® SPSS® Statistics version 24 (IBM Corp., Armonk, NY). A formal sample size calculation was not performed due to the limited availability of cadaveric and dry bone specimens. A convenience sampling method was used, whereby all available specimens meeting inclusion criteria were included in the study.

## Results

The dry bone sample included 14 individuals, consisting of 10 individuals with only unilateral femurs available and 4 individuals with bilateral femurs, for a total of 18 femur specimens. These specimens were examined for the presence and dimensions of the obturator externus groove. The analysis identified an OEG in 4 of the 10 unilateral femurs (2 deep and 2 shallow) and in 1 of the 4 bilateral sets (with a deep groove present in both femurs). This corresponds to 5 individuals (36%) with an OEG present and 6 of the 18 femur specimens (33%) with an OEG present. A summary of all groove measurements taken in the dry bone study is included in Table 1. The depth of the groove varied, with two of the femurs exhibiting a shallow groove depth of less than 1 mm. The remaining four femurs had a deeper groove, measuring more than 1 mm.

**Table 1:** Summary of Obturator Externus Groove Measurements From Dry Femur Bones Data are presented as mean ± SD and range. Descriptive statistics and Pearson correlation analysis were performed. A total of six femur bone specimens from five individuals exhibited an obturator externus groove. Percentages (%) are calculated relative to the total number of femur bones analyzed (n = 18). n: number of dry bone femur specimens, mm: millimeters, SD: standard deviation

Parameter	n (%)	Mean ± SD	Range
Groove length	6 (33)	21.35 ± 4.06 mm	14.06-25.25 mm
Groove width	6 (33)	9.02 ± 0.86 mm	7.93-10.39 mm
Groove depth - shallow (<1 mm)	2 (11)	0.8 ± 0.01 mm	0.79-0.81 mm
Groove depth - deep (>1 mm)	4 (22)	1.30 ± 0.24 mm	1.02-1.56 mm

Because most individuals in the dry bone sample were represented by only a single femur (n = 10), percentages in Table 1 are calculated relative to the total number of femur specimens (n = 18). In contrast, all cadaveric specimens included bilateral femurs, and therefore, percentages in Table 2 for the cadaveric analysis are calculated relative to the total number of individuals (n = 28). In the cadaveric sample of 28 donors, the average age at the time of death was 76 years (range: 59-95 years). Our findings revealed that six donors (two male and four female donors) exhibited a unilateral OEG. Hence, the prevalence of the groove is 21% in the cadaveric sample. Every cadaveric specimen contained an obturator externus notch. When present, the OEG started at this notch and continued for variable lengths on the posterior surface of the neck of the femurs. The depth of the groove varied. Four of the cadavers exhibited a shallow groove depth, measuring less than 1 mm. The remaining two cadavers had a deeper groove, measuring more than 1 mm, with a mean of 2.72 ± 0.70 mm. A summary of the measurements can be found in Table 2.

**Table 2:** Obturator Externus Groove Measurements From Cadaveric Femur Dissections Data are presented as mean ± SD and range. Descriptive statistics and Pearson correlation analysis were performed. A total of six cadaveric specimens exhibited an obturator externus groove. Percentages (%) are calculated relative to the total number of cadavers analyzed (n = 28). n: number of cadaveric specimens with an obturator externus groove, mm: millimeters, SD: standard deviation

Parameter	n (%)	Mean ± SD	Range
Groove length	6 (21)	35.06 ± 6.19 mm	24.38-43.55 mm
Groove width	6 (21)	9.13 ± 2.30 mm	6.57-12.67 mm
Groove depth - shallow (<1 mm)	4 (14)	0.73 ± 0.17 mm	0.52-0.92 mm
Groove depth - deep (>1 mm)	2 (7)	2.72 ± 0.70 mm	2.01-3.42 mm

**Figure 8:**
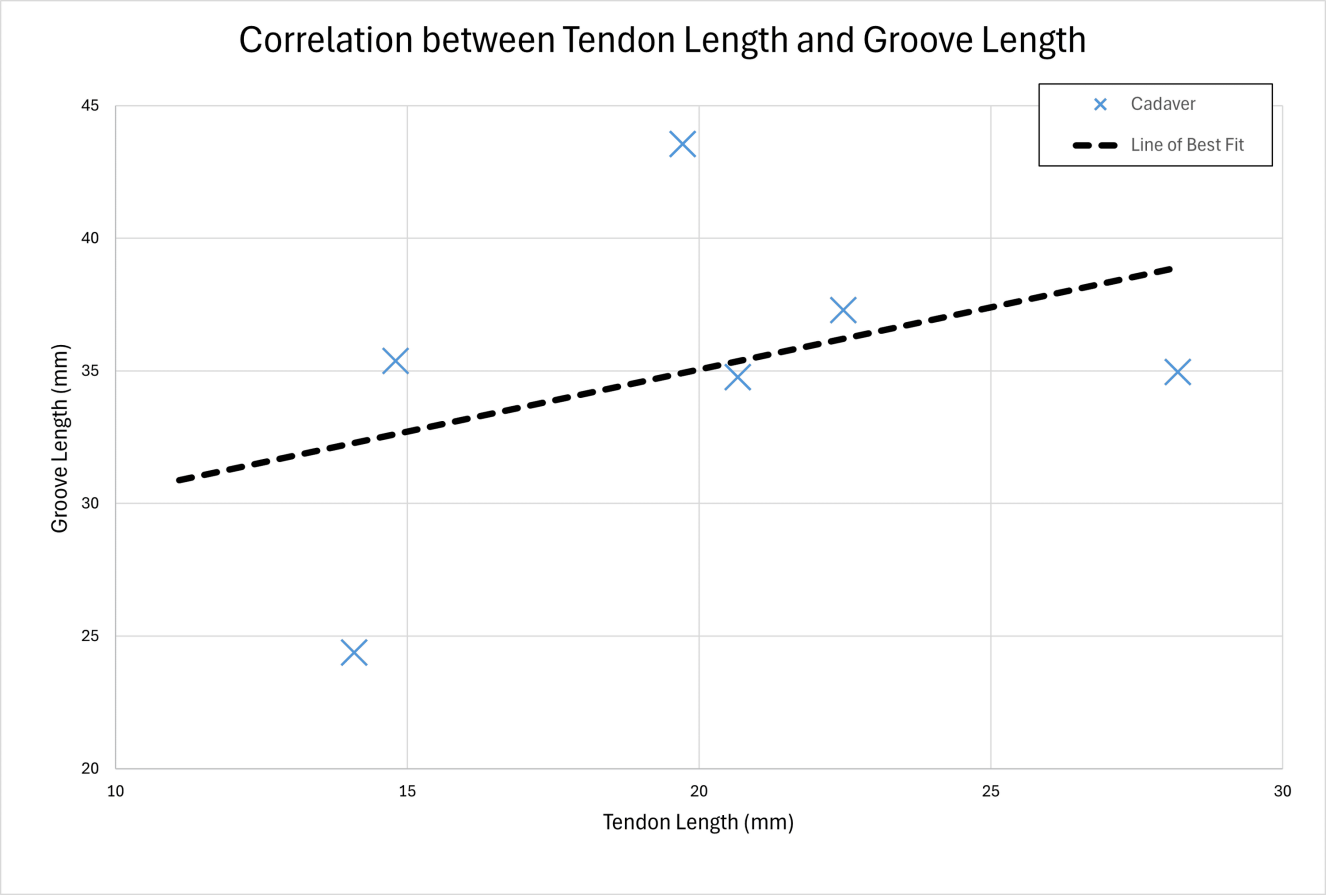
Comparison of Obturator Externus Tendon Length and Obturator Externus Groove Length in Cadavers Where a Groove Was Present This figure was created by the authors using Microsoft Excel version 2604. mm: millimeters

The obturator externus groove and the tendon length were measured in six cadaveric specimens. Among these specimens, there appeared to be a moderate positive correlation between tendon length and groove length (Pearson correlation coefficient, r = 0.395) (Figure 8). However, the statistical power was small, and the results were not statistically significant (p = 0.49).

In summary, the OEG was seen in only 36% of the dry bone sample and in 21% of the cadaveric sample. When combining the two samples (dry bone and cadaveric), the OEG in this study was seen in 11 out of the total 42 subjects, resulting in an incidence of about 26%. The obturator externus notch was consistently observed in 100% of the specimens. Additionally, the soft tissue impression accommodating the tendon up to its insertion site was observed bilaterally in all cadavers.

## Discussion

There are two predominant viewpoints regarding the OEG’s role in bipedalism in the literature [17,18]. One perspective asserts that the OEG serves as a marker for bipedalism, pointing to its presence in potential human ancestors and remains, attributed to passive contact of the OET on the femoral neck during extension, possibly occurring with upright walking [7,11,12]. An opposing view argues that the OEG merely indicates femoral extension, a trait found in various primates [14,19], including some that do not habitually extend the hip, challenging its association with bipedalism [13]. There has also been scrutiny of some fossils, such as those of *Orrorin*, as discussed by Pickford et al. in 2002 [12], regarding the significance of the OEG in inferring bipedalism [16,21]. These studies underscore the importance of a more in-depth review of the obturator externus groove in human femur specimens.

In anthropological literature, a problem contributing to the lack of understanding of the relevance of the OEG to bipedalism is the absence of agreement on its definition and naming convention. The groove has been referenced under several various names, including “intertrochanteric groove,” “gutter of obturator externus,” “external obturator footprint,” “shallow groove,” and “smooth groove” [11,14,16-20]. This has led to some confusion about its significance and prevalence among humans and early fossils. In our study, we use the term “obturator externus groove,” as first described by Day in 1969 [11]. Moreover, there are only two studies [13,22] that have examined the incidence of the OEG in humans, both of which focused on dry bone remains rather than cadaveric dissection. The methods described in the study by Lovejoy et al. identify the OEG with palpation or visually with the reflection of light and surface texture [13], both of which are subjective and prone to interobserver error.

We attempted to reproduce the methodology as described in the study by Lovejoy et al. [13] in the determination of groove presence through palpation and visualization. In addition, we categorized the grooves as shallow or deep based on the measurements of their depth. According to the results of our study, the definition of a deep groove, greater than 1 mm, corresponded with the anatomists' consensus on whether the groove was “palpable.” Grooves greater than 1 mm consistently were deemed as “palpable,” and grooves measuring less than 1 mm were considered “non-palpable.” Furthermore, the advantage of cadaveric dissection, as performed in our study, is that the OET is still visibly attached, allowing direct observation beneath it to determine the presence of the OEG and its relationship to the OET. This is unlike the dry bone specimen studies, where deterioration of bone and user interpretation may affect the findings. Additionally, our study was conducted by trained anatomists and utilized measurement tools, both of which aided in omitting user bias, and we believe contributed to more accurate findings.

Our study is the first, to the best of our knowledge, to investigate and define the obturator externus notch, soft tissue bed, and obturator externus groove in cadaveric specimens. In all specimens, we found the site of attachment of the obturator externus tendon appeared as an oval depression at the trochanteric fossa on the medial surface of the greater trochanter. The inferior wall of the trochanteric fossa appeared as a “notch” at the junction between the greater trochanter and the posterior aspect of the neck of the femur. In this study, we named this notch the “obturator externus notch” as it accommodates the terminal portion of the OET. The notch must not be confused with the OEG. As it approached its insertion site, the OET was separated from the neck of the femur by a soft tissue bed that distanced the tendon from direct contact with bone (Figures 4, 5). The soft tissue bed accommodated the OET and appeared grooved, but it did not indicate the presence of a groove on the neck of the femur. This important point highlights that without detailed anatomical knowledge, the presence of the OEG might be overestimated.

In the literature, the causation of the OEG and its relevance to bipedalism is poorly understood. Day attributes the presence of the OEG to frequent extension or hyperextension of the thigh, describing it as a “well-recognized bipedal characteristic of modern sapient femora” [11]. However, there is conflicting evidence regarding this claim. According to several studies, the obturator externus groove is present in some species of non-human primates that are not fully bipedal [9,14,15]. One of these reports suggests the groove’s presence in primates is attributed to other structural components of the hip and femur, including ischial shortening and femoral anteversion, which result in the muscle contacting the femoral neck [9]. In contrast, in some species of quadrupedal monkeys, the OEG forms despite the obturator externus tendon contacting the femoral neck during hip flexion rather than hip extension like it does in humans [14,19].

A study comparing bipedal animals found that humans are the best example of bipedalism due to biomechanical advantages, such as an erect posture [23]. Therefore, one could argue that if the OEG is a marker of bipedalism, it should be present in most or all humans. However, our findings showed that the OEG was observed unilaterally in only 21% of cadavers and 36% of dry femur bones, demonstrating that the OEG is atypical and usually seen unilaterally. This suggests that the grooves in humans and other species may result from biomechanical factors other than bipedal walking.

Using electromyographic (EMG) analysis, Stern and Larsen investigated the activity of obturator muscles in non-human primates. Their findings emphasized the significant role of the obturator externus muscle in stabilizing the hip joint and maintaining balance during climbing activities. Additionally, the presence of the obturator externus groove on the femur has been interpreted as an adaptation to accommodate the tendon of the obturator externus muscle, which is actively engaged during climbing movements. The inactivity of the obturator externus muscle during bipedal standing further supports its primary association with climbing activities rather than bipedal locomotion [9].

We propose the presence of the OEG is due to factors other than bipedalism. The six grooved cadaveric femurs and six grooved dry femurs within our sample population could be attributed to the person’s physical activities and lifestyle. We speculate that activities requiring strong hip extension, such as certain dance forms or rock climbing, may have created this groove. The unilaterality of the OEG observed in most individuals in our sample suggests that more force was applied to the dominant leg during these activities.

Another study used the presence or absence of the OEG as an independent measure for determining the mode of locomotion. They suggested that reducing the distance between the origin of the obturator externus and acetabulum increases the chance of the tendon leaving a groove on the bone [24]. We could argue that the presence of the OEG is not necessarily the result of bipedal locomotion, but from other frequent hip and femur positions that apply the obturator externus tendon to the posterior femoral neck. Obturator externus is known to be an external rotator of the hip in flexed and neutral positions and assists in adduction of a flexed hip [10]. Hence, we could speculate that frequent internal rotation of the hip and thigh abduction can stretch the muscle and result in leaving a groove.

To better understand the causation of the groove, the related soft tissue structures that surround the tendon should not be ignored. In our study, we found that the OET was cushioned by a soft tissue pad, accommodating the tendon up to its insertion site. Upon reflection of the tendon, the tendon bed appeared as a soft tissue impression bounded proximally by the ischiofemoral capsular ligament and distally by the transverse branch of the medial circumflex femoral artery, surrounded by a pad of fat. The floor of the soft tissue impression was made by the OEB and some fat covering part of the ischiofemoral capsular ligament, which separated the tendon from the posterior aspect of the femur’s neck. Although we only infused the bursae in four specimens, the filling fluid leaked into the hip joint cavity of all of them, indicating that the OEB communicates with the hip joint cavity. These anatomical observations are supported by a study that identified the OEB in 10 patients with MRI and in 1 cadaveric specimen. They hypothesized that the development of the OEB originated from the margins of the ischiofemoral capsular ligament [25], which aligns with our findings.

Various pathologies have been associated with the OEB [26,27]. It has been suggested that bursae around the hip would become pathologically involved when pressure within the joint was chronically increased and was associated with synovitis [27,28]. Hence, a groove on the posterior femur in prehistoric fossils might have resulted from hip joint disease linked to calcific obturator externus bursitis. The calcified bursa could have preserved the soft tissue impression, and pressure from the OET on the inflamed bursa might indirectly cause OEG formation on the bone cortex. Therefore, the OEG might be a result of a disease process of the bursa rather than a sign of the erect nature and bipedalism of the species.

Another potential cause of the groove could be due to repetitive, sport-specific activities or strength training. In a study on professional ballet dancers, the obturator externus muscle was significantly larger than in other trained athletes, due to repetitive external rotation [29]. As the cross-sectional area and size of muscles increase, so do the cross-sectional area and stiffness of the associated tendons, which could potentially form the OEG [30,31]. Notably, among our cadaveric donors, the groove was only found unilaterally when present. A study on fencing and badminton athletes showed that the lead extremity (the leg with greater force applied) had a significantly larger OE tendon cross-sectional area and stiffness than the non-lead extremity [32]. This could explain why the groove was mostly unilateral in our sample.

The present study contributes to the limited literature on the OEG in humans and its implications in bipedalism. Our relatively small sample size is a limitation that could be remedied by using a larger and more diverse sample size to enhance statistical power and generalize the results. Future research could use radiologic modalities to localize the obturator externus tendon and identify the groove on the back of the neck of the femur. MRI studies determining the size of the obturator externus could be advantageous, as muscle size and patient history, such as being a dancer, may correlate with the presence or absence of the OEG [29]. Moreover, radiologic assessment of the proximal femur in living subjects could correlate variables such as lifestyle, ethnicity, and demographics with the presence or absence of the obturator externus groove, providing more insight into its formation mechanism.

## Conclusions

This study investigated the incidence of the obturator externus groove and its anatomy as it relates to the obturator externus muscle tendon, surrounding soft tissue, and the femoral neck. The obturator externus groove was found to be uncommon in our specimens, leading the authors to conclude that the groove should not be used as a criterion for bipedalism. The presence of the groove might be due to other pathological, structural, or biomechanical factors.
